# Protein Profiling of Bladder Urothelial Cell Carcinoma

**DOI:** 10.1371/journal.pone.0161922

**Published:** 2016-09-14

**Authors:** Jinghai Hu, Fei Ye, Miao Cui, Peng Lee, Chengguo Wei, Yuanyuan Hao, Xiaoqing Wang, Yanbo Wang, Zhihua Lu, Matthew Galsky, Russell McBride, Li Wang, Dongwen Wang, Carlos Cordon-Cardo, Chunxi Wang, David Y. Zhang

**Affiliations:** 1 Department of Urology, The First Hospital, Jilin University, Changchun, Jilin, 130021, China; 2 Department of Pathology, Icahn School of Medicine at Mount Sinai, New York, NY, 10029, United States of America; 3 Departments of Pathology, New York University, School of Medicine, New York, NY, 10010, United States of America; 4 Division of Nephrology, Department of Medicine, Mount Sinai School of Medicine, New York, NY, 10029, United States of America; 5 Division of Hematology and Oncology, Department of Medicine, Mount Sinai School of Medicine, New York, NY, 10029, United States of America; 6 Departments of Genetics and Genomic Sciences, Mount Sinai School of Medicine, New York, NY, 10029, United States of America; 7 Department of Urology, First Hospital of Shanxi Medical University, Taiyuan, Shanxi, 030002, China; University of Oklahoma Health Sciences Center, UNITED STATES

## Abstract

This study aimed to detect protein changes that can assist to understand the underlying biology of bladder cancer. The data showed forty five proteins were found to be differentially expressed comparing tumors vs non-tumor tissues, of which EGFR and cdc2p34 were correlated with muscle invasion and histological grade. Ten proteins (ß-catenin, HSP70, autotaxin, Notch4, PSTPIP1, DPYD, ODC, cyclinB1, calretinin and EPO) were able to classify muscle invasive BCa (MIBC) into 2 distinct groups, with group 2 associated with poorer survival. Finally, 3 proteins (P2X7, cdc25B and TFIIH p89) were independent factors for favorable overall survival.

## Introduction

Bladder cancer (BCa) is among the five most common malignancies worldwide. In the United States, an estimated 74,690 new cases of bladder cancer were diagnosed in 2014, with 15,580 deaths expected [1]. More than 90% of bladder cancers are urothelial cell carcinomas, 5% are squamous cell carcinomas, and less than 2% are adenocarcinomas [2]. Previous studies showed that bladder cancer is characterized by heterogeneous subtypes, i.e. non-muscle-invasive and muscle-invasive, showing variable clinical presentation, genetic background, treatment implications, and prognosis. Non-muscle-invasive disease (70% of newly diagnosed bladder cancer cases) can generally be managed without surgical removal of the bladder, frequently recurs, but in the absence of progression to more invasive disease is generally not life threatening, while muscle-invasive lesions (30% of cases) are associated with worse outcomes [3]. Owing to the high recurrence rate and the lifelong surveillance, bladder cancer is one of the most expensive cancers to treat [4].

For patients with muscle-invasive disease, the gold standard of therapy is radical cystectomy with regional lymphadenectomy [5, 6]. While such treatment may be curative, approximately 50% of patients will develop lethal metastatic recurrence [7]. Due to these poor outcomes, the integration of perioperative systemic chemotherapy has been explored in the treatment of MIBC [8]. Randomized clinical trials have demonstrated that neoadjuvant cisplatin-based combination chemotherapy is associated with an improvement in survival and has been integrated as a standard treatment approach. However, the absolute survival benefit with neoadjuvant chemotherapy is modest and many patients are exposed to the potential side effects of treatment without clinical benefit. Because of the heterogeneity in outcomes of patients with MIBC treated with surgery alone, and the benefit achieved in only a subset of patients with the integration perioperative chemotherapy, there has been an intense interest in the development of prognostic and predictive biomarkers (for both surgery and chemotherapy) as well as novel therapeutic targets [9, 10].

Recent integrated genomic studies [11, 12] have revealed complex genomic alterations in MIBC including recurrent somatic mutations, copy number variations (CNV), translocations, and mRNA and miRNA expression changes. The most common recurrent somatic mutations were found in TP53, MLL2, ARID1A, PIK3CA, RB1, HRAS and FGFR3 genes. The most common CNVs were seen in CDKN2A, E2F3SOX4, CCND1, RB1 and EGFR genes. Based on the gene expression patterns, MIBC can be grouped into 2-4 subtypes, including papillary-like, epithelial lineage, basal/squamous-like, and ERBB2 [13, 14]. Integrated genomic analysis revealed that several important pathways are involved in the pathogenesis of MIBC including p53/RB pathway (93%), histone modification pathway (89%), RTK/Ras/PIK3CA pathway (72%) and SWI/SNF pathway (64%). While these studies and others have markedly advanced our genomic understanding of MIBC, the proteomic landscape of MIBC has not been fully explored.

To understand the global alterations in signaling protein expression and activation, we investigated the expression of 285 proteins and phosphoproteins in bladder urothelial cell carcinoma tissues and adjacent non-tumor tissues by means of Protein Pathway Array (PPA) method. PPA is a recently developed high-throughput protein assay, which combines multiplex immunoblotting with computational analysis. PPA can characterize hundreds of proteins in tissue samples and identify alterations in protein expression [15–17]. Through PPA, we identified several protein changes that correlate with the clinical outcomes and found the canonical pathways that were most significant to MIBC based on the protein expression profile.

## Materials and Methods

### Patients and tissue samples

This study included 74 patients with bladder urothelial carcinoma who underwent surgery at The First Hospital of Jilin University, Jilin Province, China between April, 2009 and February, 2013. Among them, 17 cases were treated by transurethral resection, 4 cases were treated by partial cystectomy, and 53 cases were treated by radical cystectomy. The research is approved by the Institutional Review Board (IRB) of Jilin University. The participants provided their written informed consent to participate in this study. The ethics committees/IRBs approve this consent procedure.

Forty-three pairs of BCa and adjacent non-tumor mucosa (30 in the training set and 13 in the validation set) and an additional 31 cancer tissues (non-tumor adjacent tissues were not available) were obtained. The representative tumors and adjacent non-tumor tissues were dissected and frozen in liquid nitrogen after immediate pathological examination. Tumor samples of 3×3×5 mm were taken from areas without gross necrosis and adjacent non-tumor mucosa samples of 3×3×5 mm were taken from the same specimens approximately 3 cm away from the tumor margin. The tumor samples did not contain non-tumor mucosal tissue. The adjacent non-tumor mucosa samples contained mucosa and a portion of adherent submucosa; neither tumor nor dysplasia was included. The clinicopathological characteristics of the patients are summarized in Table 1.

**Table 1:** The clinicopathological characteristics of 74 patients.

Clinicopathological characteristics	No. of patients (%), n = 74
Age (years)	
≤60	26 (35.1)
>60	48 (64.9)
Gender	
Male	62 (83.8)
Female	12 (16.2)
Smoking history	
No	27 (36.5)
Yes	47 (63.5)
Histological grade	
Low grade	23 (31.1)
High grade	51 (68.9)
Vascular invasion	
Presence	21 (28.4)
Absence	53 (71.6)
Tumor size (cm)	
≦3	18 (24.3)
>3	56 (75.7)
Multifocality	
Single	26 (35.1)
Multiple	48 (64.9)
pT classification	
Ta	4 (5.4)
T1	31 (41.9)
T2	21 (28.4)
T3	13 (17.6)
T4	5 (6.8)

The median follow-up was 25.2 months (range, 4–61 months). The last day of follow-up was July 16, 2014. The survival time was counted from the date of surgery to the last day of follow-up or the date of death of any cause. The cause of death of the majority of the patients was cancer recurrence or distant metastasis. However, patients who died of other causes or were lost to follow-up were also included in the analysis (ie, overall survival).

### Protein Pathway Array analysis

Total proteins were extracted from the 117 fresh frozen samples (43 tumor/ non-tumor pairs, and 31 tumor only) by adding 1× cell lysis buffer (Cell Signaling Technology, Danvers, MA) with protease and phosphatase inhibitor cocktail (Roche Applied Science, Indianapolis, IN). The lysate was sonicated 3 times for 15 seconds each time and then centrifuged at 14,000 rpm for 30 minutes at 4°C. The protein concentration was determined by the BCA Protein Assay kit (PIERCE, Rockford, IL), as described previously[16].

Approximately 300 μg of protein lysate was loaded in one well across the entire width of a 10% SDS polyacrylamide gel and separated by electrophoresis. After electrophoresis, the proteins were transferred electrophoretically to a nitrocellulose membrane (Bio-Rad, Hercules, CA), which was then blocked for 1 hour in blocking buffer including either 5% milk or 3% bovine serum albumin in 1×Tris-HCI, NaCl, and Tween 20 (TBST) containing 20 mmol/L Tris-HCl (pH 7.5), 100 mmol/L NaCl, and 0.1% Tween-20.Then the membrane was clamped on a Western blotting manifold (Mini-PROTEAN II Multiscreen Apparatus, Bio-Rad, Hercules, CA) that isolates 20 channels across the membrane. The multiplex immunoblot was performed using a total of 285 protein-specific or phosphorylation site-specific antibodies (S1 Table). The antibodies were divided into 9 sets, each set contained 26–36 antibodies. All antibodies (from various companies) were validated independently before inclusion in the PPA. For the first set of 36 primary antibodies, a mixture of 2 antibodies in the blocking buffer were added to each channel and then incubated at 4°C overnight. The membrane was then washed with 1×Tris-buffered saline and 1×Tris-buffered saline-Tween-20, and was further incubated with secondary anti-rabbit (Bio-Rad), anti-mouse (Bio-Rad), or anti-goat (Santa Cruz Biotechnology, Santa Cruz, CA) antibody conjugated with horseradish peroxidase for 1 hour at room temperature. The membrane was developed with chemiluminescence substrate (Immun-Star HRP Peroxide Buffer/Immun-Star HRP Luminol Enhancer, Bio-Rad), and chemiluminescent signals were captured using the ChemiDoc XRS System (Bio-Rad). The same membrane was then stripped off using stripping buffer (Restore Western blot stripping buffer, Thermo Scientific, Rockford, IL) and then used to detect a second set of 36 primary antibodies, as described above. Each membrane can be blotted with 3 sets of antibodies.

For PPA data analysis, the signals of each protein were determined by densitometric scanning (Quantity One software package, Bio-Rad) and the background was locally subtracted from raw protein signal. Then the background subtracted intensity was normalized by the “global median subtraction” method to reduce variation among different experiments. Specifically, the intensity of each protein from each sample was divided by the total intensities of all proteins from the same sample and then multiplied by average intensities of all proteins in all samples [17].

### Statistical analysis

The significant analysis of microarray (SAM) tool http://www-stat.stanford.edu/tibs/SAM) and paired t-test were used to identify the proteins differentially expressed between tumors and non-tumor tissues. Overlap genes with p < 0.05 from t-test and q < 5% from SAM test were selected for downstream analysis. K-fold cross-validation (K = 10) was used to select those with greatest power to distinguish tumors from non-tumor tissues. K-fold cross validation was performed using BRB Array Tools software, version 3.3 (http://linus.nci.nih.gov/BRB-ArrayTools.html). Multi Experiment Viewer was used to perform the unsupervised hierarchical clustering analysis. Protein expression profiling in different pT classifications were mapped using grid analysis of time-series expression (GATE, version 1.4.1; http://amp.pharm.mssm.edu/maayanlab/gate.htm). IBM SPSS statistics 17 software (Armonk, NY) was used to plot Kaplan–Meier curves and perform Cox proportional hazard regression analysis to test potential associations between protein expression and overall survival. A p value < 0.05 was considered to be statistically significant.

### Signaling network analysis

The discriminating genes of the corresponding proteins identified by PPA were imported into IPA (Version 9.0, www.ingenuity.com) for network analysis. IPA uses the Ingenuity Knowledge Base as a reference set and identifies local networks that are particularly enriched for the input genes by computational algorithms. Furthermore, IPA uses a Fisher’s exact test to determine which pathways (i.e. canonical pathways and biological functions) are significantly linked to the input gene set compared with the whole Ingenuity knowledge base. This analysis was to attempt to explore the relationship between the different signaling pathways and the clinical factors in BCa, and further analysis and correlate these pathways.

## Results

### Differentially expressed signaling proteins

**Fig 1:**
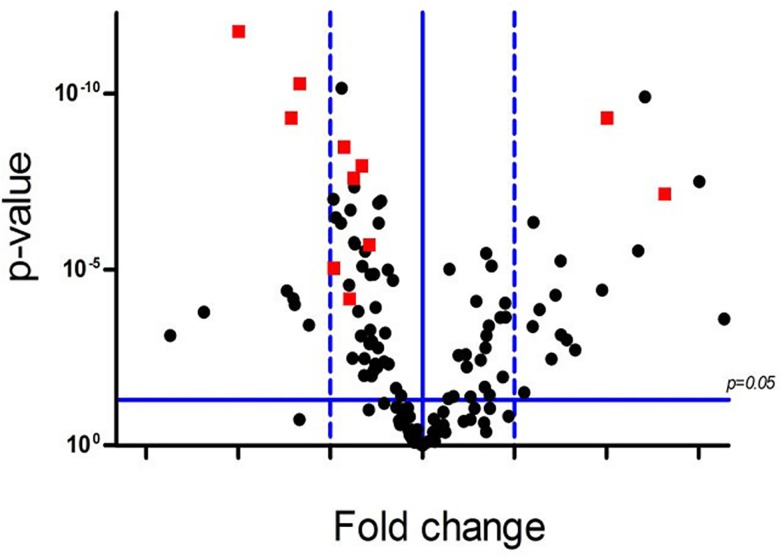
The volcano plot depicts the fold and p-value differences in expression levels of 136 proteins between the tumor and non-tumor tissues. The horizontal blue line indicates p = 0.05 with points above the line having p < 0.05.

**Fig 2:**
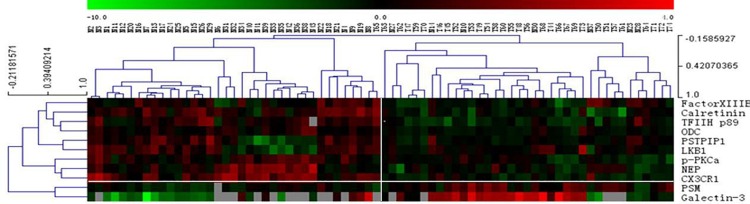
Hierarchical clustering analysis of 11 differentially expressed proteins in 31 tumors (T) and 43 non-tumor (N) samples. The color scale showed the level of expression. Red indicates overexpression, green indicates underexpression, black indicates no change, and gray no expression. The number in each column represents the sample number. Each row represents a protein.

There were 136 of 285 proteins detected in either tumor or non-tumor tissues (Fig 1), and 45 of 136 proteins were found to be differentially expressed between tumors and non-tumor tissues in all 43 paired samples based on the paired t-test and SAM analysis (t-test p<0.05 or SAM test q<5%, S2 Table). Among them, 22 proteins and phosphoproteins were upregulated in tumors, including HIF-3α, HDAC1, IL-1ß, Bad, Mesothelin, cyclinD1, PDEF, XIAP, ß-catenin, HMG-1, p27, MDM2, p-RB, Maspin, MetRS, HSP70, cdc2 p34, PCNA, p38ß, PSM, CHK1, and Galectin-3. In contrast, 23 proteins and phosphoproteins were downregulated in tumors, including Factor XIIIB, p-P44/42, Calretinin, Notch4, TFIIHp89, Autotaxin, NFkBp50, PSTPIP1, E-Selectin, WT1, p-PKCa, Bak, CX3CR1, DPYD, ADH, NEP, p44/42MAPK, LKB1, ODC, cdc42, cPKCα, GLP-1R and HES1. To identify a best set of proteins to separate benign tissues and BCa, all paired samples were randomly divided into either training set or validation set. We carried out supervised K-fold cross validations (K = 10) using two class prediction models, including a support vector machine (SVM) and 3-nearest neighbor (3NN). Eleven proteins (PSTPIP1, TFIIHp89, Factor XIIIB, PSM, NEP, Calretinin, Galectin-3, LKB1, ODC, p-PKCα, CX3CR1) with the value of p< 0.01 were selected as markers for classification (red squares in Fig 1). To further confirm the ability of these eleven proteins to classify bladder cancer, we tested these proteins using additional 31 tumors and original 43 non-tumor samples with the two-way hierarchical clustering analysis (Fig 2), which revealed distinct patterns for the two groups although several samples were misclassified.

### Correlation of protein expression with clinical characteristics of BCa

To examine the relationship between protein expression and clinical characteristics of BCa (i.e., muscle invasive, histological grade, vascular invasion, tumor size, multifocality, sex and age), we performed the unpaired t test and SAM analysis (p<0.05 or q<5%). Table 2 shows the results of differentially expressed proteins with statistical significance between tumors and non-tumor tissues based on the clinicopathological characteristics. For example, EGFR and cdc2 p34 were correlated with muscle invasion and histological grade. In contrast, no correlations were found between protein expression and gender (Male vs Female) or age (≦60 vs >60).

**Table 2:** Correlations of protein expression with clinicopathological characteristics.

Clinicopathological	Differential expression protein
Muscle invasion (Yes vs No)	EGFR↑, cdc2p34↑, Bcl-xL↑, Cytokeratin19↑, MDM2↑
Histological grade (High vs Low)	EGFR↑, cdc2 p34↑
Vascular invasion (Yes vs No)	Calretinin↑, V-ATPase H↑, HSP 70↓, p27↓, ASC↓, VSV-G tag↓, DRG1↓, Maspin↓
Tumor Size (>3 vs ≦3 cm)	Stat 3↓, Rho A↓
Tumor Multifocality (Multiple vs Single)	FGF-8↓, p-Stat3↓, PDEF↓, NEP↓, Bcl-xL↓

Note: An upward arrow indicates an increased protein expression and a down arrow indicates a decreased protein expression.

### Correlation of canonical pathways with clinic parameters

**Fig 3:**
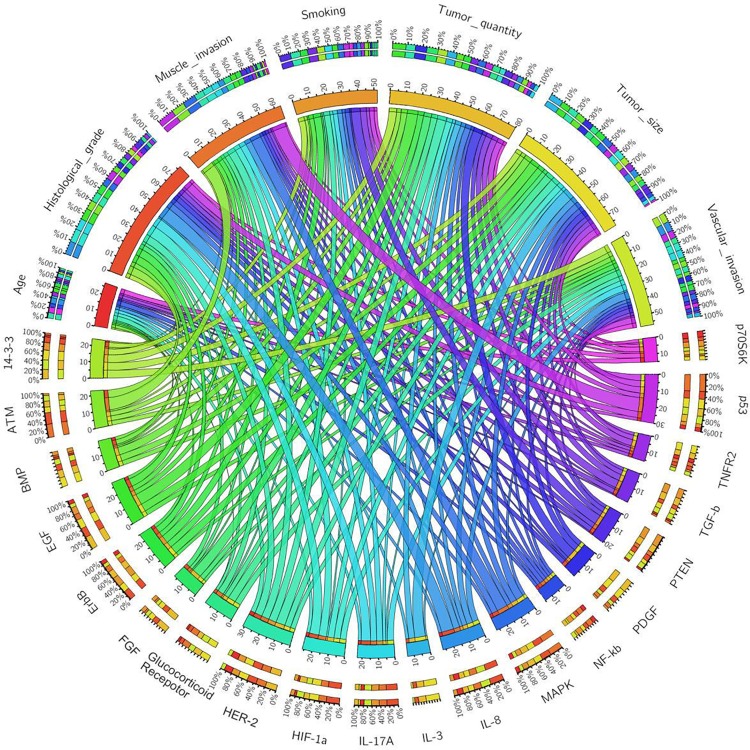
The circos plot depicts the relationship between the different signaling pathways and the clinicopathological categories in BCa. Each signaling pathway and clinicopathological category are assigned with a specific color. The association between the clinicopathological categories and signaling pathways are depicted by the arcs. The area of each colored ribbon depicts the proportion of the signaling pathway contributes to a particular clinicopathological category.

Data of the differentially expressed proteins in each clinicopathological category was uploaded into IPA for functional annotation and pathway analysis. The most relevant canonical signaling pathways enriched with these differentially expressed proteins were generated and ranked by IPA. The top 20 signaling pathways were p53 (-log p = 8.72), ATM (-log p = 7.49), MAPK (-log p = 6.78), HER-2 (-log p = 4.95), HIF1α(-log p = 4.57), IL-17A (-log p = 4.01), IL-8 (-log p = 3.81), glucocorticoid receptor (-log p = 3.36), EGF (-log p = 3.31), ErbB (-log p = 2.93), PTEN (-log p = 2.66), NF-κB (-log p = 2.34), TNFR2 (-log p = 1.76), BMP (-log p = 1.35), PDGF (-log p = 1.35), FGF (-log p = 1.3), TGF-β(-log p = 1.29), 14-3-3 (-log p = 1.17), p70S6K (-log p = 1.16) and IL-3 (-log p = l.11).Then we applied the circos plot to depict the relationship between the different signaling pathways and the clinicopathological categories in BCa which included muscle invasion, histological grade, smoking, vascular invasion, tumor size and tumor multifocality (Fig 3). In this plot, age used as a reference since our study demonstrated no correlation between protein expression and patients’ age. The plot showed that p53, MAPK, and ATM signaling pathways may play a key role in MIBC. In high grade BCa, IL-8, IL-17A and HIF-1α may be the most important signaling pathways. Among all twenty canonical signaling pathways, P53 and HER2 occupy the maximal proportions of the distribution, suggesting that they may be the most important pathways in BCa.

### Association between differentially expressed proteins and tumor T stages

**Fig 4:**
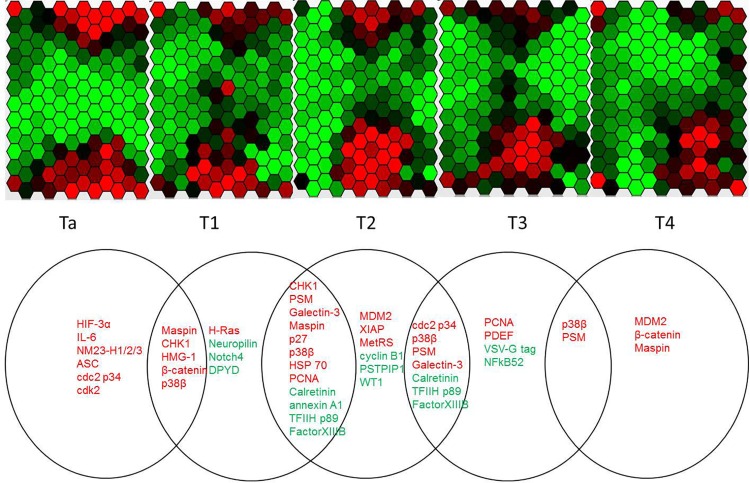
Differentially expressed proteins in different T stages. a) The hexagonal array displays the changing patterns of the proteins expression from Ta to T4. Each hexagon represents a protein, the color of a hexagon reflects its level of expression in current T stage with respect to corresponding non-tumor tissue. b) The significantly expressed proteins between tumor and non-tumor tissues in different T stages were listed in their own circle which represents a specific stage. The proteins listed in the overlap area were shared by 2 adjacent stages. Proteins in red are upregulated and proteins in green are downregulated.

To understand the protein expression changes among different clinical stages, the unpaired t test (p<0.05) was performed to identify the proteins expressed differentially between tumors and non-tumor tissues at each T stage. There were 11, 18, 19, 11, and 5 differentially expressed proteins in Ta, T1, T2, T3, and T4, respectively (Fig 4, S3–S7 Tables). In order to profile the protein expression more comprehensively and systematically, the protein expression results of all tumor samples was uploaded into GATE [18], and the systems-level molecular regulatory dynamics was created (S1 File (Video)).

### Sub-classification of Muscle Invasive Bladder Cancer

**Fig 5:**
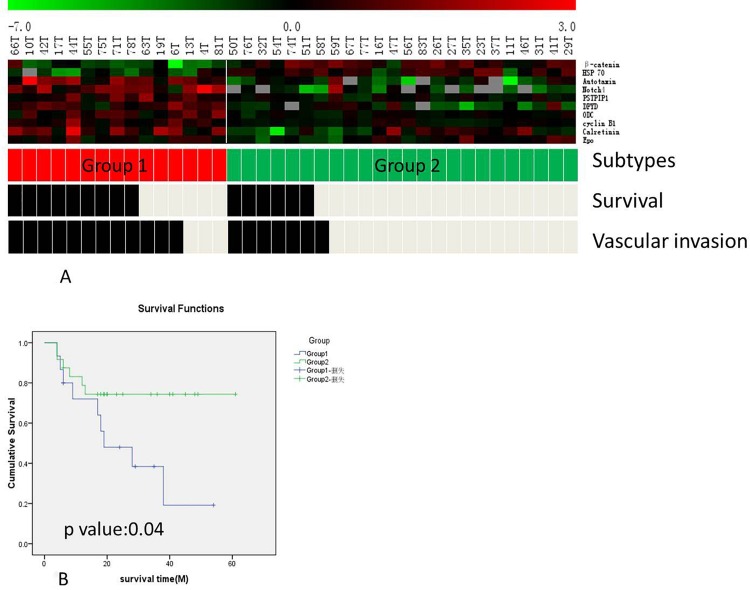
(a) Hierarchical clustering analysis of 10 differentially expressed proteins in MIBC tumors. 39 MIBC cases were clustered into 2 groups with group 1 had a higher mortality and vascular invasion rates. (b) The Kaplan–Meier and log-rank survival analysis showed that the group 1 was associated with a poorer prognosis than group 2 (p = 0.04).

To further examine the molecular difference among MIBC, we carried out supervised K-fold cross validation (K = 10) using BRB Array Tools software. Ten proteins (ß-catenin, HSP70, Autotaxin, Notch4, PSTPIP1, DPYD, ODC, cyclinB1, Calretinin and EPO) with p value <0.01 were selected to classify MIBC into 2 distinct groups (Fig 5A). Group 1 was enriched with activation of Notch4 pathway, while Group 2 enriched with beta catenin pathway. Importantly, each group had different clinical outcomes. In group 1, there were 9 deaths (60%) and 12 vascular invasion cases (80%) out of 15 cases. In group 2, there are 6 deaths (25%) and 7 vascular invasion cases (30%) out in 24 cases (Fig 5A). Furthermore, group 1 had much poorer survival than group 2 based on the Kaplan–Meier analysis (p = 0.04, Fig 5B).

### Association between protein expression and overall survival in MIBC

**Fig 6:**
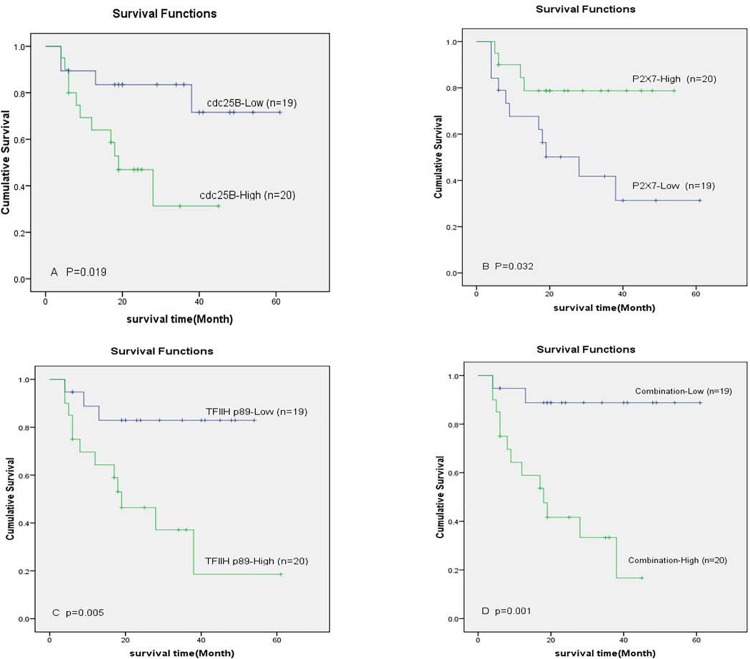
The Kaplan–Meier and log-rank survival analysis showed that the group with low level expression of P2X7 and high level expression cdc25B and TFIIH p89 was associated with a poorer prognosis (p = 0.032, p = 0.019, and p = 0.005, respectively) and risk score could stratify patients more accurately (p = 0.001).

To identify proteins that can predict overall survival, an univariate Cox proportion hazard regression analysis was performed on 136 expressed proteins in 39 MIBC cases. Eight proteins (cyclinB1, cdc25B, Calretinin, Rab7, FGF8, TFIIHp89, IL18 and P2X7) were statistically associated with overall survival (Table 3). Clinicopathological characteristics were also included in univariate Cox proportional hazards regression analysis and the vascular invasion was found to be the only factor correlated with overall survival (Table 3). Then the 8 proteins and vascular invasion were included in a multivariate Cox proportional hazards regression analysis and cdc25B, TFIIHp89, and P2X7 were confirmed to be independent prognostic markers. To clarify the predictive ability of the 3 proteins, samples were separated into either high or low expression groups based on the median protein expression. The groups with low level expression of P2X7 and high level expressions of cdc25B and TFIIH p89 were associated with a poorer prognosis (p = 0.032, p = 0.019, and p = 0.005, respectively, Fig 6). These data suggest that cdc25B, TFIIHp89, and P2X7 can be used to stratify patients with MIBC. In order to generate a single risk assessment measure, risk score was calculated by combining these proteins for each patient as described previously [17]. Those with higher risk scores survival poorer than those with lower risk scores (Fig 6, p = .001).

**Table 3:** Results of uni-variate Cox Proportional HR analyses.

	P value	HR	95.0% CI	
			Lower	Upper
vascular invasion	0.045	3.224	1.024	10.147
cyclinB1	0.005	3.360	1.447	7.802
cdc25B	0.010	7.197	1.610	32.161
Calretinin	0.026	1.709	1.067	2.738
Rab7	0.019	0.656	0.461	0.933
FGF8	0.014	0.367	0.165	0.819
TFIIHp89	0.003	2.450	1.360	4.415
IL18	0.035	0.111	0.014	0.857
P2X7	0.014	0.301	0.116	0.785

Statistically significant as an independent predictor of survival (p<0.05)

95% CI, 95% confidence interval; HR, hazard ratio.

## Discussion

To understand the genetic basis of BCa, many efforts are being derived from gene expression microarray studies [12, 19, 20]. But so far, a comprehensive catalog of genetic alterations is far from complete. In our study, we took a different approach to identify protein alterations using the Protein Pathway Array technology [15]. Of 285 proteins tested, 45 were differentially expressed between non-tumor tissues and tumors, suggesting that significant dysregulation of signaling proteins in tumor. These differentially expressed proteins are important in diverse cellular processes, including cell cycle, DNA damage repair and inflammation. Based on IPA analysis and circos plot, these differentially expressed proteins are involved in p53, HER2, MAPK and ATM signaling pathways. Our data further confirmed that bladder cancer is a heterogeneous tumor and many different pathways are involved in bladder cancer pathogenesis.

Some of the dysregulated proteins were reported in bladder cancer in previous studies, consistent with our findings. For example, PSM and Galectin-3 were up-regulated in bladder cancer and those patients with high level expressions had a worse outcome [21, 22]. Calretinin was down-regulated in transitional cell carcinoma of the urinary bladder [23]. However, the expression of some of the proteins differed from that described in previous publications. For example, the expressions of p-PKCα and ODC (ornithine decarboxylase) were decreased in our study, but the increased levels of PKCα and ODC expression were reported by other groups [24, 25]. The discrepancy could be due to different antibodies used (phosphorylated PKCα vs total PKCα) or different methodology used (immunoblot vs immunohistochemistry for ODC). Finally, some of the dysregulated proteins found in this study have not been reported previously in bladder cancer, including NEP, Factor XIIIB, PSTPIP1, TFIIHp89 and CX3CR1. These findings demonstrate the ability of using Protein Pathway Array method to identify new markers in bladder cancer.

In this study we found that several proteins correlate with clinical behaviors of BCa, suggesting the potential use of these proteins as clinical biomarkers. For examples, EGFR and cdc2 p34 were associated with muscle invasion and histological grade. Overexpression of EGFR in bladder cancer has been widely reported and several studies have shown EGFR positivity to be associated with high tumor stage, tumor progression, and poor clinical outcome [26, 27]. cdc2 p34 is reportedly involved in the tumor proliferation, apoptosis, invasion and metastasis, and overexpressed in a variety of tumors, including the bladder cancer [28]. Our results also showed the expressions of Bcl-xL, cytokeratin 19 and MDM2 were increased in MIBC, consistent with previous reports.

For muscle-invasive bladder cancer, the standard treatment is radical cystectomy followed by urinary diversion. However, quality of life of postoperative patients is poor [29]. It is well known that even among patients who have similar clinical and pathological features, the outcome varies. Therefore, it may be beneficial to identify accurate prognostic predictors in order to select those patients with aggressive MIBC for radical cystectomy and spare those with less aggressive MIBC for more conservative treatment. In this study, we were able to classify 39 MIBC tumors into two groups, based on 10 differentially expressed proteins, and each group had different clinical outcomes with a poorer survival in group 2. It is worthy to note that NFkB pathway was activated in group 1, while in group 2, the signaling pathways were enriched in regulation of apoptosis, cell transformation and proliferation. The results suggested that the differences between these two groups at molecular level and group 2 had more profound alterations in cellular functions.

In order to predict outcomes of MIBC, we further identified 3 proteins (cdc25B, TFIIHp89, and P2X7) as independent predictors of overall survival. Based on the expression levels of these proteins and their associations with survival, a risk score can be further calculated for each individual patient with MIBC for more accurate prognosis. Cdc25B is a phosphatase that activates the cyclin dependent kinase CDC2 by removing two phosphate groups. Cdc25B is required for cells to enter into mitosis. Overexpression of Cdc25B was found in many malignancies including bladder, gastric, endometrial, and renal cell cancers [30, 31], and cdc25B overexpression was associated with a higher recurrence rate and a poorer prognosis in bladder cancer [32]. TFIIH p89 (XPB), encoded by ERCC3 gene, is part of the TFIIH transcription factor complex which is involves in the transcription of RNA polymerase I and II and the repair of damaged DNA [33]. ERCC1 may predict survival in bladder cancer patients treated with platinum-based therapy and a low ERCC1 level was associated with better survival [34]. However, so far there is no report on the expression level of ERCC3 (TFIIH p89) in bladder cancer. The receptor P2X7 is membrane-bound, ligand-gated cation channel in response to ATP binding. P2X7 receptors have been implicated in ATP-mediated cell death, regulation of receptor trafficking, and inflammation [35, 36]. Overexpression of P2X7 has been reported in chronic lymphocytic leukemia, breast cancer, prostate cancer and neuroblastoma [37–40]. However, the expression of P2X7 in bladder cancer and its association with clinical outcomes have not been reported previous.

In conclusion, our data showed a broad dysregulation of signaling proteins in BCa, suggesting the important roles of these signaling proteins in carcinogenesis. The altered expression of some of the proteins correlated with invasion and metastasis, whereas other proteins correlated with overall survival, indicating that different sets of signaling proteins associate with different tumor behaviors and clinical outcomes. Future studies will be focused on understanding the roles of these proteins in controlling tumor behaviors. Due to the small sample size, the clinical utility of these proteins needs to be confirmed in a different cohort of patients.

## Supporting Information

S1 File (video). The systems-level molecular regulatory dynamics of protein expression results. (PDF)

S1 Table List of antibodies included in the Protein Pathway Array(285). (DOCX)

S2 Table Proteins differentially expressed between tumor and non-tumor tissues. (DOCX)

S3 Table Proteins differentially expressed between tumor and non-tumor tissues in Ta stage. (DOCX)

S4 Table Proteins differentially expressed between tumor and non-tumor tissues in T1 stage. (DOCX)

S5 Table Proteins differentially expressed between tumor and non-tumor tissues in T2 stage. (DOCX)

S6 Table Proteins differentially expressed between tumor and non-tumor tissues in T3 stage. (DOCX)

S7 Table Proteins differentially expressed between tumor and non-tumor tissues in T4 stage. (DOCX)
