# Supplementary figures and images for: Protein Profiling of Bladder Urothelial Cell Carcinoma
Source: PLoS One. 2016 Sep 14;11(9):e0161922. doi: 10.1371/journal.pone.0161922 (PMC5023150; doi:10.1371/journal.pone.0161922)

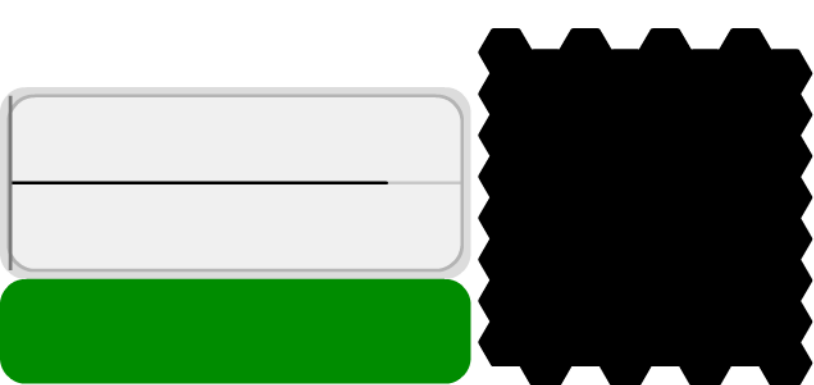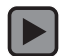

Supplement: S1 File — (PDF) [file pone.0161922.s001.pdf]
