# Supplementary material for: Protein Profiling of Bladder Urothelial Cell Carcinoma
Source: PLoS One. 2016 Sep 14;11(9):e0161922. doi: 10.1371/journal.pone.0161922 (PMC5023150; doi:10.1371/journal.pone.0161922)
Supplement: S1 Table — (DOCX) [file pone.0161922.s002.docx]

| **S1 Table. List of antibodies included in the Protein Pathway Array(285)** |
| --- |
|  |
| **Antibodies specific for phosphorylated signal proteins** |
| p-PKCα^Ser657^, p-PDK1^Ser241^, p-PKCα/βII^Thr638/641^, p-p53^Ser392^, p-AKT^Ser473^, p-PTEN^Ser380^,  p-RB^Ser780^, p-β-catenin^Ser33/37/Thr41^, p-c-Jun^Ser73^, p-STAT3^Ser727^, p-P44/42 MAPK(Erk1/2)^Thr202/Tyr204^, p-GSK-3α/β^Ser21/9^, p-p70 S6 kinase^Thr389^, p-EIF4B^Ser422^, p-MET ^Tyr1234^, p-Smad^Ser463/465^, p-ERK5^Thr218/Tyr220^, p-p90RSK^Ser380^, p-CREB^Ser133^, p-PKCδ^Thr505^, p-FAK^Tyr397^, p-CDC2^Tyr15^,p-STAT5^Tyr694^, p-RB^Ser807/811^, p-p38^Thr180/Tyr182^ |
| **Antibodies specific for non-phosphorylated signal proteins** |
| Fas, FOXM1, ERα, SYK, MetRS, Twist, Lyn, KLF6, CaMKKa, SK3, Stat1, CyclinB1, CyclinD1, CDK6, CDC25B, EGFR, CDK2, p27, TDP1, CDK4, HER2/ErbB2, 14-3-3β, cPKCα, CyclinE, SLUG, CDC25C, HSP90, CHK1, MDM2, CDC2p34, E2F-1, PCNA , p63, p38β, Rap1, β-catenin, p44/42 MAPK (Erk1/2), Akt, XIAP, Bcl-2, Patched, HIF-1α, HIF-2α,TTF-1，p53, Notch4, PTEN, SRC-1, Eg5, HIF-3α, Bax, N-cadherin, TNF-α, CDC42, EIF4B, Vimentin, OPN, Survivin, E-cadherin, TGF-β, ERβ, WT1, Mesothelin, VEGF, ATF-1, Ep-CAM, Bad, NFκB p52, NFκB p50, Calretinin, IL-1β, H-Ras, Bcl-6, K-Ras, NFκB p65, CREB, BID, Maspin, DRG1 , Factor XIII B, IGFBP5, HCAM, ICAM-1, c-Flip, PSM, Rab7, VCAM-1, FGF-8, NEP, Bcl-xL, Endoglin, Bak, TFIIH p89, Nkx-3.1, RIP, NM23-H1/2/3,c-IAP2, Epo, PDEF, Stat 3, ERCC1, uPAR, KAI1, L-Selectin, PSCA, E-Selectin ,Cytokeratin 5, Cytokeratin 19, Pax-2, Raf-B, Cytokeratin18, E2A, Glutamine Synthetase, HMG-1, ASCL1, JAK2, Jagged1, Aurora A/AIK, Keratin 10, VSV-G tag, FKHR, FAH, Axin, HES1, FTα, Flt-3/ Flk-2, PERK, IL-3Rα, BECN1, CD33, Wnt-1, HDAC1, uPA, NFATc1, TCF-1,α-tubulin, MTHFR, P504S, MSR, DHFR, PC2 , Tyro3 , cSHMT, LSD1 , MTHFD 2 , MTHFD 1, MAT IIβ, SOD-1, ODC , LKB1, Hint1 , HSL, PEDF, NHERF-2, SPAK, NMT1, FEN-1, ADH, Dnmt1, IDO, GLP-1R, PIk, MetAP-2, COL1A2, PRL3, SMAD4, MTA1, MMP2, Snail1, COX-2, P-cadherin, connexin43, IL-6, MMP9, HGF, MMP7, p16, ADAM10, CUL-1, TIMP3, RHAMM, Annexin A1, RegⅣ,ADAM8, EphB2, SMAD7, fusin, FUS /TLS, CKR7, FGFR4, RUNX3, BMP-2, p14, KISS1, TIP30, RAGE, NALP1, IL-18, CTGF, CTLA-4, ASC, TIRAP, IL-11, c-Fms/CSF-1R, tPA, VAP-1, MMP-13, AIM2, IL-8RA, Caspase-1 p10, Autotaxin, CX3CR1, CathepsinB, P2X7, IRF-1, Integrinα4, SUGT1, Pannexin-1,CARD12, PSTPIP1, NOS2, RANKL, Tak1, IFN-γ, IL-8, TRAF6, DARPP-32, ITF, β3 Tubulin, Calpain 2, HSP 70, PrP, Galectin-3, V-ATPase H, p73, NQO1, Cdx2, IGF-Irβ, Tau, TNF-R2, YB-1, GSTP1, P-JNK, Rho A, PKC ε, HoxC11, tsg 101, ALG-2, HNF-3α, Calpastatin, TS, DACH1, HSP 27, MGr1-Ag, JNK1, DPYD, Clusterin, DDB2, Ribosomal Protein L6. |

Underlines indicate detectable expression in either tumors or adjacent normal tissues.

β-Actin and GAPDH serve as internal control.

All phosphorylation state-specific antibodies were obtained from Cell Signaling Technology (Danvers, MA) except the following antibodies: 1) p-PKCα (Ser657) was purchase from Upstate Biotech (Lake Placid, NY); 2) p-Met (Tyr1234) and p-FAK(Tyr397) were purchased from Santa Cruz Biotechnology (Santa Cruz, CA).

All non-phosphorylated antibodies were obtained from Santa Cruz Biotechnology (Santa Cruz, CA), except the following antibodies: 1) Stat1, HER2/ErbB2, β-Catenin, p44/42 MAPK (Erk1/2), Akt, Notch4, eIF4B, NFκB p50, CREB, Estrogen Receptor α, Bcl-xL, RIP, Aurora A/AIK, MMP-9 and Snail were obtained from Cell Signaling Technology (Danvers, MA); 2) XIAP and Glutamine Synthetase were obtained from BD Biosciences (San Jose, CA); 3) TGF-β was obtained from R&D Systems (Minneapolis, MN); 3) Hsp90 was obtained from ENZO Life Sciences (Farmingdale, NY); 4) HIF-2α was obtained from Novus Biologicals (Littleton, CO); 4) Cytokeratin 18 was obtained from Dako Corporation (Carpinteria, CA); 5) FAH was obtained from Proteintech Group (Chicago, IL); 6) Keratin 10 was obtained from Covance Research Products (Berkeley, CA); 7) VSV-G were obtained from Abcam Corporation (Cambridge, MA).
