# Supplementary material for: Protein Profiling of Bladder Urothelial Cell Carcinoma
Source: PLoS One. 2016 Sep 14;11(9):e0161922. doi: 10.1371/journal.pone.0161922 (PMC5023150; doi:10.1371/journal.pone.0161922)
Supplement: S2 Table — (DOCX) [file pone.0161922.s003.docx]

**S2 Table. Proteins differentially expressed between tumor and non-tumor tissues.**

|  | **Protein** | **Gene ID** | **Tumor**  **-Average** | **Normal**  **-Average** | **Fold Change** | **t-test**  **p-value(%)** | **SAM-test**  **q-value(%)** |
| --- | --- | --- | --- | --- | --- | --- | --- |
| up | p-RB | RB1 | 354.04 | 154.28 | 2.29 | 0.01 | 0.00 |
|  | MetRS | MARS | 641.98 | 304.43 | 2.11 | 0.17 | 0.00 |
|  | cyclin D1 | CCND1 | 5243.54 | 3494.52 | 1.50 | 0.00 | 0.00 |
|  | p27 | CDKN1B | 767.45 | 450.44 | 1.70 | 0.03 | 0.00 |
|  | CHK1 | CHEK1 | 3775.74 | 783.36 | 4.82 | 0.00 | 0.00 |
|  | MDM2 | MDM2 | 700.87 | 283.56 | 2.47 | 0.07 | 0.00 |
|  | cdc2 p34 | CDC2 | 628.47 | 172.24 | 3.65 | 0.05 | 0.00 |
|  | PCNA | PCNA | 4462.45 | 1181.14 | 3.78 | 0.00 | 0.00 |
|  | p38ß | MAPK14 | 770.79 | 192.58 | 4.00 | 0.00 | 0.00 |
|  | ß-catenin | CTNNB1 | 1453.28 | 697.71 | 2.08 | 0.84 | 0.00 |
|  | XIAP | XIAP | 542.34 | 241.59 | 2.24 | 0.28 | 0.39 |
|  | HIF-3α | HIF3A | 551.20 | 381.09 | 1.45 | 0.87 | 0.74 |
|  | Mesothelin | MSLN | 1744.75 | 933.39 | 1.87 | 1.02 | 0.39 |
|  | Bad | BAD | 649.46 | 403.57 | 1.61 | 0.20 | 0.00 |
|  | IL-1ß | IL1B | 591.46 | 366.96 | 1.61 | 0.23 | 0.00 |
|  | Maspin | SERPINB5 | 1009.84 | 356.58 | 2.83 | 0.00 | 0.00 |
|  | PSM | FOLH1 | 3513.96 | 898.69 | 3.91 | 0.00 | 0.00 |
|  | PDEF | SPDEF | 1066.41 | 593.04 | 1.80 | 0.09 | 0.00 |
|  | HMG-1 | HMGB1 | 351.00 | 65.21 | 5.38 | 0.00 | 0.00 |
|  | HDAC1 | HDAC1 | 963.35 | 667.45 | 1.44 | 0.49 | 0.39 |
|  | HSP 70 | HSPA1A | 1440.11 | 657.32 | 2.19 | 0.00 | 0.00 |
|  | Galectin-3 | LGALS3 | 1931.11 | 416.47 | 4.64 | 0.00 | 0.00 |
| down | p-PKCa | PRKCA | 776.98 | 1443.69 | 0.54 | 0.03 | 0.00 |
|  | p-P44/42 | MAPK3 | 474.88 | 1181.14 | 0.40 | 0.00 | 0.00 |
|  | cPKCa | PRKCB1 | 5369.08 | 8438.82 | 0.64 | 0.11 | 0.00 |
|  | p44/42 MAPK | MAPK3 | 2355.99 | 4045.29 | 0.58 | 0.00 | 0.00 |
|  | Notch4 | NOTCH4 | 412.01 | 951.90 | 0.43 | 0.02 | 0.00 |
|  | cdc42 | CDC42 | 1292.79 | 1871.53 | 0.69 | 0.15 | 0.00 |
|  | WT1 | WT1 | 1408.75 | 2217.10 | 0.64 | 0.01 | 0.00 |
|  | NFkBp50 | NFKB1 | 855.15 | 1296.00 | 0.66 | 0.00 | 0.00 |
|  | Calretinin | CALB2 | 834.08 | 2101.70 | 0.40 | 0.00 | 0.00 |
|  | FactorXIIIB | F13B | 238.90 | 953.22 | 0.25 | 0.00 | 0.00 |
|  | NEP | MME | 653.96 | 1096.72 | 0.60 | 0.00 | 0.00 |
|  | TFIIH p89 | ERCC3 | 1276.95 | 3428.28 | 0.37 | 0.00 | 0.00 |
|  | Bak | BAK1 | 3843.06 | 6085.52 | 0.63 | 0.03 | 0.00 |
|  | E-Selectin | SELE | 588.97 | 1087.81 | 0.54 | 0.00 | 0.00 |
|  | HES1 | HES1 | 357.63 | 524.22 | 0.68 | 0.03 | 0.00 |
|  | ODC | ODC1 | 1665.75 | 2547.10 | 0.65 | 0.00 | 0.00 |
|  | LKB1 | STK11 | 1720.71 | 2718.55 | 0.63 | 0.00 | 0.00 |
|  | ADH | ADH1A | 1331.48 | 2309.06 | 0.58 | 0.00 | 0.00 |
|  | GLP-1R | GLP1R | 912.44 | 1332.60 | 0.68 | 0.03 | 0.00 |
|  | Autotaxin | ENPP2 | 349.54 | 535.87 | 0.65 | 0.72 | 0.00 |
|  | CX3CR1 | CX3CR1 | 579.55 | 979.29 | 0.59 | 0.08 | 0.00 |
|  | PSTPIP1 | PSTPIP1 | 1210.61 | 2181.48 | 0.55 | 0.00 | 0.00 |
|  | DPYD | DPYD | 1370.50 | 1870.34 | 0.73 | 0.49 | 0.00 |

Up means the expressions of these proteins were up-regulated in tumor samples; down means the expressions of these proteins were down-regulated in tumor samples.
