# Supplementary material for: Protein Profiling of Bladder Urothelial Cell Carcinoma
Source: PLoS One. 2016 Sep 14;11(9):e0161922. doi: 10.1371/journal.pone.0161922 (PMC5023150; doi:10.1371/journal.pone.0161922)
Supplement: S3 Table — (DOCX) [file pone.0161922.s004.docx]

**S3 Table. Proteins differentially expressed between tumor and non-tumor tissues in Ta stage.**

|  | **Protein** | **Gene ID** | **Tumor**  **-Average** | **Normal**  **-Average** | **Fold Change** | **t-test**  **p-value(%)** | **SAM-test**  **q-value(%)** |
| --- | --- | --- | --- | --- | --- | --- | --- |
| **up** | CHK1 | CHEK1 | 3244.54 | 783.36 | 4.14 | 1.38 | 0.00 |
|  | cdc2 p34 | CDC2 | 497.67 | 172.24 | 2.89 | 2.68 | 0.00 |
|  | p38ß | MAPK14 | 1255.19 | 192.58 | 6.52 | 1.35 | 0.00 |
|  | ß-catenin | CTNNB1 | 2372.99 | 697.71 | 3.40 | 0.41 | 0.00 |
|  | HIF-3a | HIF3A | 2003.00 | 381.09 | 5.26 | 0.02 | 0.00 |
|  | NM23-H1/2/3 | NME1 | 3271.93 | 1140.10 | 2.87 | 0.32 | 0.00 |
|  | ASC | PYCARD | 2148.02 | 770.69 | 2.79 | 0.32 | 0.00 |
|  | IL-6 | IL6 | 334.43 | 105.83 | 3.16 | 0.00 | 0.00 |
|  | Maspin | SERPINB5 | 2328.13 | 356.58 | 6.53 | 0.00 | 0.00 |
|  | Cdk2 | CDK2 | 2038.30 | 838.55 | 2.43 | 0.91 | 0.00 |
|  | HMG-1 | HMGB1 | 462.08 | 65.21 | 7.09 | 0.18 | 0.00 |
