# Supplementary material for: Protein Profiling of Bladder Urothelial Cell Carcinoma
Source: PLoS One. 2016 Sep 14;11(9):e0161922. doi: 10.1371/journal.pone.0161922 (PMC5023150; doi:10.1371/journal.pone.0161922)
Supplement: S4 Table — (DOCX) [file pone.0161922.s005.docx]

**S4 Table. Proteins differentially expressed between tumor and non-tumor tissues in T1 stage.**

|  | **Protein** | **Gene ID** | **Tumor**  **-Average** | **Normal**  **-Average** | **Fold Change** | **t-test**  **p-value(%)** | **SAM-test**  **q-value(%)** |
| --- | --- | --- | --- | --- | --- | --- | --- |
| **up** | CHK1 | CHEK1 | 4134.50 | 783.36 | 5.28 | 0.00 | 0.00 |
|  | Galectin-3 | LGALS3 | 2307.95 | 416.47 | 5.54 | 0.00 | 0.00 |
|  | PSM | FOLH1 | 3445.68 | 898.69 | 3.83 | 0.00 | 0.00 |
|  | Maspin | SERPINB5 | 1743.26 | 356.58 | 4.89 | 0.00 | 0.00 |
|  | p27 | TP27 | 1111.42 | 450.44 | 2.47 | 0.00 | 0.00 |
|  | p38β | MAPK14 | 757.83 | 192.58 | 3.94 | 0.00 | 0.00 |
|  | HSP 70 | HSPA1A | 1801.87 | 657.32 | 2.74 | 0.00 | 0.00 |
|  | H-Ras | HRAS | 482.42 | 169.76 | 2.84 | 0.03 | 0.00 |
|  | PCNA | PCNA | 3682.04 | 1181.14 | 3.12 | 0.07 | 0.00 |
|  | β-catenin | CTNNB1 | 1761.38 | 697.71 | 2.52 | 0.09 | 0.00 |
|  | HMG-1 | HMGB1 | 354.40 | 65.21 | 5.44 | 0.10 | 0.00 |
| **down** | Calretinin | CALB2 | 738.91 | 2101.70 | 0.35 | 0.00 | 0.00 |
|  | annexin A1 | ANXA1 | 662.50 | 1528.66 | 0.43 | 0.00 | 0.00 |
|  | TFIIH p89 | ERCC3 | 1314.80 | 3428.28 | 0.38 | 0.00 | 0.00 |
|  | neuropilin | NRP1 | 551.93 | 1230.75 | 0.45 | 0.00 | 0.00 |
|  | FactorXIIIB | F13B | 358.64 | 953.22 | 0.38 | 0.00 | 0.00 |
|  | Notch4 | NOTCH4 | 207.09 | 951.90 | 0.22 | 0.00 | 0.00 |
|  | DPYD | DPYD | 1180.36 | 1870.34 | 0.63 | 0.05 | 0.00 |
