# Supplementary material for: Protein Profiling of Bladder Urothelial Cell Carcinoma
Source: PLoS One. 2016 Sep 14;11(9):e0161922. doi: 10.1371/journal.pone.0161922 (PMC5023150; doi:10.1371/journal.pone.0161922)
Supplement: S5 Table — (DOCX) [file pone.0161922.s006.docx]

**S5 Table. Proteins differentially expressed between tumor and non-tumor tissues in T2 stage.**

|  | **Protein** | **Gene ID** | **Tumor**  **-Average** | **Normal**  **-Average** | **Fold Change** | **t-test**  **p-value(%)** | **SAM-test**  **q-value(%)** |
| --- | --- | --- | --- | --- | --- | --- | --- |
| **up** | CHK1 | CHEK1 | 3666.19 | 783.36 | 4.68 | 0.00 | 0.00 |
|  | cdc2 p34 | CDC2 | 1034.79 | 172.24 | 6.01 | 0.00 | 0.00 |
|  | p38β | MAPK14 | 985.08 | 192.58 | 5.12 | 0.00 | 0.00 |
|  | PCNA | PCNA | 5024.34 | 1181.14 | 4.25 | 0.00 | 0.00 |
|  | HSP 70 | HSPA1A | 2383.91 | 657.32 | 3.63 | 0.00 | 0.00 |
|  | PSM | FOLH1 | 2561.04 | 898.69 | 2.85 | 0.00 | 0.00 |
|  | Galectin-3 | LGALS3 | 1611.35 | 416.47 | 3.87 | 0.01 | 0.00 |
|  | MDM2 | MDM2 | 934.55 | 283.56 | 3.30 | 0.00 | 0.00 |
|  | XIAP | XIAP | 821.73 | 241.59 | 3.40 | 0.01 | 0.00 |
|  | Maspin | SERPINB5 | 1147.98 | 356.58 | 3.22 | 0.00 | 0.00 |
|  | MetRS | MARS | 739.74 | 304.43 | 2.43 | 0.07 | 0.00 |
|  | p27 | TP27 | 1023.89 | 450.44 | 2.27 | 0.01 | 0.00 |
| **down** | Calretinin | CALB2 | 576.55 | 2101.70 | 0.27 | 0.00 | 0.00 |
|  | cyclin B1 | CCNB1 | 5875.54 | 13911.95 | 0.42 | 0.00 | 0.00 |
|  | TFIIH p89 | ERCC3 | 960.70 | 3428.28 | 0.28 | 0.00 | 0.00 |
|  | annexin A1 | ANXA1 | 648.78 | 1528.66 | 0.42 | 0.00 | 0.00 |
|  | PSTPIP1 | PSTPIP1 | 929.34 | 2181.48 | 0.43 | 0.00 | 0.00 |
|  | FactorXIIIB | F13B | 337.19 | 953.22 | 0.35 | 0.00 | 0.00 |
|  | WT1 | WT1 | 1044.92 | 2217.10 | 0.47 | 0.00 | 0.00 |
