# Supplementary material for: Protein Profiling of Bladder Urothelial Cell Carcinoma
Source: PLoS One. 2016 Sep 14;11(9):e0161922. doi: 10.1371/journal.pone.0161922 (PMC5023150; doi:10.1371/journal.pone.0161922)
Supplement: S6 Table — (DOCX) [file pone.0161922.s007.docx]

**S6 Table. Proteins differentially expressed between tumor and non-tumor tissues in T3 stage.**

|  | **Protein** | **Gene ID** | **Tumor**  **-Average** | **Normal**  **-Average** | **Fold Change** | **t-test**  **p-value(%)** | **SAM-test**  **q-value(%)** |
| --- | --- | --- | --- | --- | --- | --- | --- |
| **up** | PCNA | PCNA | 4647.67 | 1181.14 | 3.93 | 0.00 | 0.00 |
|  | cdc2 p34 | CDC2 | 800.57 | 172.24 | 4.65 | 0.00 | 0.00 |
|  | Galectin-3 | LGALS3 | 1749.09 | 416.47 | 4.20 | 0.03 | 0.00 |
|  | p38β | MAPK14 | 768.54 | 192.58 | 3.99 | 0.01 | 0.00 |
|  | PSM | FOLH1 | 2448.78 | 898.69 | 2.72 | 0.00 | 0.00 |
|  | PDEF | SPDEF | 1409.55 | 593.04 | 2.38 | 0.01 | 0.00 |
| **down** | TFIIH p89 | ERCC3 | 1130.88 | 3428.28 | 0.33 | 0.00 | 0.00 |
|  | FactorXIIIB | F13B | 296.28 | 953.22 | 0.31 | 0.00 | 0.00 |
|  | Calretinin | CALB2 | 1045.11 | 2101.70 | 0.50 | 0.02 | 0.00 |
|  | VSV-G tag | VSV-G | 848.86 | 1599.44 | 0.53 | 0.02 | 0.00 |
|  | NFkB52 | NFKB2 | 741.41 | 1351.66 | 0.55 | 0.02 | 0.00 |
