# Supplementary material for: Protein Profiling of Bladder Urothelial Cell Carcinoma
Source: PLoS One. 2016 Sep 14;11(9):e0161922. doi: 10.1371/journal.pone.0161922 (PMC5023150; doi:10.1371/journal.pone.0161922)
Supplement: S7 Table — (DOCX) [file pone.0161922.s008.docx]

**S7 Table. Proteins differentially expressed between tumor and non-tumor tissues in T4 stage.**

|  | **Protein** | **Gene ID** | **Tumor**  **-Average** | **Normal**  **-Average** | **Fold Change** | **t-test**  **p-value(%)** | **SAM-test**  **q-value(%)** |
| --- | --- | --- | --- | --- | --- | --- | --- |
| **up** | PSM | FOLH1 | 3457.73 | 898.69 | 3.85 | 0.74 | 0.00 |
|  | β-catenin | CTNNB1 | 1813.67 | 697.71 | 2.60 | 1.39 | 0.00 |
|  | MDM2 | MDM2 | 966.26 | 283.56 | 3.41 | 1.44 | 0.00 |
|  | Maspin | SERPINB5 | 705.67 | 356.58 | 1.98 | 0.72 | 0.00 |
|  | p38β | MAPK14 | 1075.60 | 192.58 | 5.59 | 0.90 | 0.00 |
